# A single batch synthesis of pure phase Mo_2_C from ammonium molybdate: pathway and properties

**DOI:** 10.55730/1300-0527.3735

**Published:** 2025-04-08

**Authors:** Melek CUMBUL ALTAY

**Affiliations:** Department of Metallurgical and Materials Engineering, Faculty of Engineering, İstanbul University-Cerrahpaşa, İstanbul, Turkiye

**Keywords:** Ammonium molybdate tetrahydrate, chemical vapor deposition, hydrogen, methane, molybdenum carbide, reduction

## Abstract

This study presents an original, effective, and environmentally friendly method for synthesizing pure molybdenum carbide (Mo_2_C) from ammonium molybdate tetrahydrate (AMT) without generating carbon dioxide, a greenhouse gas. The process involves the sequential transformation of AMT to Mo_2_C, which follows the reaction pathway of (NH_4_)_6_Mo_7_O_24_→MoO_3_→MoO_2_→Mo→Mo_2_C. This transformation is achieved by strategically altering the gas atmosphere, switching from Ar to H_2_ at 800 K and then from H_2_ to CH_4_ at 1000 K. Thermal analysis, X-ray diffraction (XRD), and scanning electron microscopy (SEM) techniques were used to characterize AMT and the products. Mass measurements were used to follow the conversion of AMT to intermediate products and to the final product (Mo_2_C). It was found that 57.67% of AMT was converted to Mo_2_C, in agreement with the theoretical value (57.74%). Differential scanning calorimetry/thermogravimetry curves revealed four steps at 401 K, 495 K, 507 K, and 595 K during AMT decomposition to MoO_3_. XRD patterns revealed the formation of phase-pure Mo_2_C, with characteristic diffraction peaks 2θ = 34.176°, 2θ = 37.712°, and 2θ = 39.197° assigned to the (100), (002), and (101) crystal planes, respectively. SEM images showed that fine Mo_2_C particles with a thickness of 0.1 μm was obtained from very coarse AMT particles (>50 μm). In order to determine the solid and gaseous phases likely to form during the reaction, thermodynamic analysis using Gibbs’ free energy minimization method was also carried out prior to synthesis. The reduction reactions and the resulting morphologies of the synthesized materials were discussed in terms of thermodynamic results and density changes associated with the conversions. This study demonstrates a novel reaction pathway that sequentially converts the molybdenum species from Ammonium Molybdate Tetrahydrate (AMT) to the final Mo_2_C phase without the release of CO_2_.

## 1. Introduction

In the 21^st^ century, the environmental impact of greenhouse gas emissions has become a growing concern, intensifying the need for eco-friendly approaches in material synthesis [1]. Developing innovative methods to minimize the release of greenhouse gases, such as methane and carbon dioxide, has emerged as an increasingly crucial priority. Recently, numerous studies have focused on exploring alternatives to carbon-based reductants like coal and natural gas and have investigated the use of hydrogen as a means to reduce greenhouse gas emissions, particularly in energy-intensive industries like iron and steel manufacturing [2].

Molybdenum carbide (Mo_2_C) is a highly versatile material that offers a wide range of promising applications across various fields. Its exceptional physical properties, such as high hardness (1800 kgf/mm^2^), high melting point (2960 K), and exceptional chemical stability, make it an attractive additive for the development of hard coatings and wear-resistant components [3]. Moreover, Mo_2_C has demonstrated significant potential in thermoelectric and electrochemical catalysis, as well as energy storage applications, making it a sought-after material in the development of advanced technologies and devices [4].

Traditionally, Mo_2_C has been produced through the carburization of molybdenum powders using solid carbon as the carbon source. This process typically requires elevated temperatures ranging from 1673 K to 1773 K [5]. However, the use of solid carbon reactants necessitates such high temperatures, which has prompted researchers to explore alternative synthesis methods that can potentially operate at lower temperatures or with different carbon precursors.

In recent years, researchers have explored the use of gaseous carbon-containing precursors, such as methane, as alternative carbon sources for the synthesis of molybdenum carbide (Mo_2_C). These gaseous precursors offer potential advantages over traditional solid carbon sources, as they can enable the synthesis of Mo_2_C at lower temperatures and potentially reduce the emissions of greenhouse gases like carbon dioxide during the production process. For instance, Mo_2_C has been synthesized from MoO_3_ at 900 K using an H_2_-CH_4_ gas mixture [6]. The production of Mo_2_C powders typically involves the reaction of MoO_3_ with gaseous reactants comprising a mixture of H_2_ and hydrocarbons [6–9].

The use of methane and other hydrocarbon gases as carbon sources has been investigated to develop more environmentally friendly and energy-efficient methods for the fabrication of this versatile ceramic material [10]. However, this direct reaction often leads to the generation of harmful gases such as CO and CO_2_, which contribute to the greenhouse effect. The methods used to synthesize Mo_2_C are not environmentally friendly or cost-effective. To mitigate these environmental concerns, direct reactions of hydrocarbons and CO with MoO_3_ should be avoided in the fabrication of Mo_2_C. Therefore, the synthesis of Mo_2_C from AMT necessitates the implementation of intricate and costly multi-step procedures. In a recent study on the synthesis of pure Mo_2_C by a one-pot technique with AMT as the starting material and hexamethylenetetramine (C_6_H_12_N_4_) as the reducing agent, the AMT/hexamethylenetetramine molar ratio was set at 1:8, the reaction temperature was maintained at 1073 K, and the reaction time was set at 8 h. These experimental parameters were employed in a specially designed autoclave. It has been reported that in the carburization of MoO_2_ with C_6_H_12_N_4_, the formation of H_2_O, CO_2_, and N_2_ gases has been observed as gaseous products in addition to the reaction product Mo_2_C [11].

This study presents an innovative approach to produce pure phase molybdenum carbide (Mo_2_C) from ammonium molybdate tetrahydrate as precursor material in a shorter reaction time using an efficient single batch synthesis method with the aim of minimizing greenhouse gas emission by preventing CO and CO_2_ gas formation. The key aspect of this approach is that it does not generate carbon dioxide (CO_2_) as a byproduct during the synthesis process. Previous research has shown that gaseous carbon-containing compounds, such as methane, can be used as potential carbon sources for Mo_2_C synthesis. Additionally, using hydrogen as a reducing agent has been demonstrated to minimize the formation of greenhouse gases like CO_2_. Building upon these earlier findings, the study presents a novel reaction pathway that sequentially converts the molybdenum species from AMT to the final Mo_2_C phase without the release of CO_2_.

## 2. Materials and methods

AMT (H_24_Mo_7_N_6_O_24_.4H_2_O) powder with a purity of 99.98%, supplied by Sigma Aldrich, was used as the Mo source. The hot-wall furnace employed for the experiments consisted of Fe-Cr-Al alloy heating elements, a quartz tube (diameter 20 mm × length 50 mm), gas lines (Ar, H_2_, and CH_4_), and gas flow meters. Prior to the experiment, the furnace was cleaned and purged with Ar to remove any residues that may lead to CO_2_ formation. An alumina boat filled with AMT powder (0.15 g) was heated at a rate of 15 K/min to 800 K under an Ar flow (42.5 standard cubic centimeters per minute, sccm). The gas was then switched to H_2_ (370 sccm) at 800 K, followed by heating at 15 K/min from 900 K to 1000 K. After holding for 15 min in H_2_, only CH_4_ flowed (13.4 sccm) for 45 min at 1000 K. This was followed by furnace cooling under Ar flow (42.5 sccm). The experiment was interrupted at points B, C, and Dto characterize the intermediate products.

The extents of the reactions were calculated by


(1) $$\text{Mass }(\%)=({\text{m}}_{\text{p}}/{\text{m}}_{\text{o}})\times 100,$$

where m_p_ is the product mass and m_o_ is the original AMT mass. Mass measurements were conducted at room temperature via an electronic balance (Sartorius) with a sensitivity of ±10^−4^ g. Differential Scanning Calorimetry (DSC) coupled with Thermogravimetric Analysis (TGA) curve of AMT powder (51.168 mg) was determined using equipment (Perkin Elmer STA6000 model) in dried air at a flow rate of 100 sccm during heating at a rate of 10 K/min to 1000 K. X-ray diffraction (XRD) analysis was used to identify phases via a Rigaku D/Max-2200/PC instrument with a Cu radiation tube (λ K_α1_ = 0.15406 nm). Morphologies were examined via a scanning electron microscope (SEM) (FEI Quanta FEG250) operated at 5 kV under low vacuum.

A computer program utilizing the Gibbs free energy minimization method [12,13] was employed to forecast the compositions in the Mo-O-H (MoO_3_-H_2_) and Mo-C-H (Mo-CH_4_) system’s condensed and gaseous phases at a specified temperature, input composition, and pressure (1 atm). Previous to computation, a separate input file was prepared for each temperature. Gibbs free energy values for species are derived from the thermochemical tables [14,15]. An input data file for the Mo-O-H system contained 14 gaseous species (e.g., Mo, MoO_2_, MoO_3_, H_2_MoO_4_, H, H_2_, H_2_O, H_2_O_2_, O_2_) and 6 solid (Mo, MoO_2_, MoO_3_, Mo_4_O_11_, Mo_8_O_23_, and Mo_9_O_26_) components at temperatures ranging from 800 to 1000 K. The input data file for the Mo-C-H system included 39 gaseous species (e.g., Mo, CH, CH_2_, CH_3_, CH_4_, C_2_H, C_2_H_2_) and 4 solid phases (Mo, C, MoC, and Mo_2_C) at a temperature of 1000 K.

## 3. Results and discussion

### 3.1. Thermodynamic analysis

**Figure 1:**
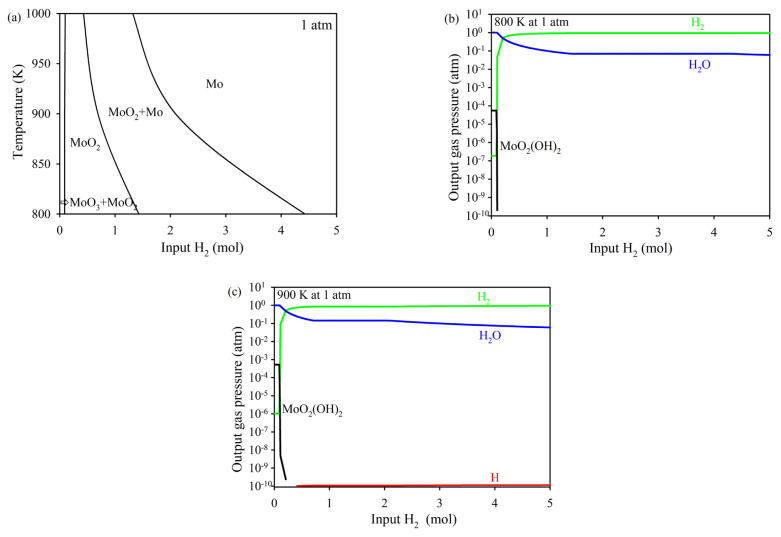
(a) Equilibrium solid phase diagram computed in the MoO_3_-H_2_ system as a function of temperature and input H_2_ content. Variations of the partial pressures of the species with the input H_2_ content at (b) 800 K, (c) 900 K, and (d) 1000 K.

Thermodynamic analysis provides a fundamental understanding of phase transformations and reaction equilibria in the synthesis process. This analysis is crucial for determining the optimal conditions required to achieve the desired Mo and Mo_2_C phase. In this study, thermodynamic calculations were conducted to examine the reduction and carburization steps in the Mo-O-H and Mo-C-H systems. The equilibrium states of solid and gaseous phases were predicted at different temperatures and gas compositions, offering valuable insights into the reaction mechanisms that govern the formation of pure-phase Mo_2_C [12,13]. Figure 1a illustrates the equilibrium solid phase diagram computed in the Mo-O-H system, showing the phase stability as a function of temperature and input H_2_ content. Four solid phase regions are revealed, which are MoO_3_+MoO_2_, MoO_2_, MoO_2_+Mo, and Mo. The reduction of molybdenum oxide to Mo is complete under the H_2_ atmosphere at various H_2_ amounts, including over 4.43 atm at 800 K and over 1.32 atm at 1000 K. Figures 1b–d display the variations in the partial pressures of gaseous species at (b) 800 K, (c) 900 K, and (d) 1000 K as a function of input H_2_ content. As a result of the calculations, only gas components with a partial pressure of over 10^−10^ atm are considered for the graphical representation of the gas components in the system. Previous experimental studies on the volatilization of molybdenum in water vapor atmospheres have suggested the formation of gaseous hydrated molybdenum species. Suggested and necessary major volatilization reactions in the literature are given in Eqs. 2–4[14,16].


(2) $${\text{MoO}}_{3}(\text{s})+{\text{H}}_{2}\text{O}(\text{g})\to {\text{MoO}}_{2}{\left(\text{OH}\right)}_{2}(\text{g})$$

(3) $${\text{MoO}}_{2}(\text{s})+2{\text{H}}_{2}\text{O}(\text{g})\to {\text{MoO}}_{2}{\left(\text{OH}\right)}_{2}+{\text{H}}_{2}(\text{g})$$

(4) $${\text{MoO}}_{2}(\text{s})+2{\text{H}}_{2}\text{O}(\text{g})\to {\text{MoO}}_{3}.{\text{H}}_{2}\text{O}+{\text{H}}_{2}(\text{g})$$

As seen in Figure 1b–c, the abundance of both H_2_ and H_2_O remains notably high across the studied temperature range, as indicated by the green and blue curves. The thermodynamic calculations indicate that the formation of gaseous MoO_2_(OH)_2_ species increases as the temperature rises, as indicated by the black curve. As the temperature increases, the thermodynamic stability of MoO_2_(OH)_2_ shifts, leading to an increase in its partial pressure under equilibrium conditions. This trend is supported by Gibbs free energy minimization method, which indicates that MoO_2_(OH)_2_ formation becomes more favorable at higher temperatures within the given reaction atmosphere. Additionally, its presence is influenced by the reduction pathways of MoO_3_ and MoO_2_, where hydrogen plays a critical role in determining the intermediate species present in the system. For example, at temperatures of 800 K and 1000 K, the partial pressures of MoO_2_(OH)_2_ are around 5.58 × 10^−6^ atm and 3.18 × 10^−3^ atm, respectively, with H_2_ gas at 0.09 atm. These results align well with previous experimental findings, which suggest that the presence of water vapor enhances the volatility of molybdenum by forming hydrated gas-phase species such as MoO_2_(OH)_2_ and MoO_3_.H_2_O [17–19]. Moreover, the relevant molybdenum gas species is identified as H_2_MoO_4_ in the JANAF thermochemical tables. However, in line with previous experimental studies, this species will be referred to as MoO_2_(OH)_2_ throughout this manuscript to ensure consistency with observed volatilization mechanisms. Figures 1c–1d also shows that the partial pressure of H gas is significantly lower, as indicated by the red curve.

**Figure 2:**
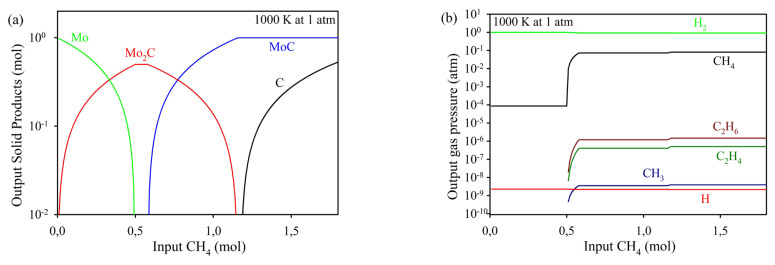
(a) Equilibrium solid phase diagram computed in the Mo-CH_4_ system as a function of input CH_4_ content, (b) variations of partial pressures of the gaseous products with the input CH_4_ content at 1000 K.

Figure 2a presents the equilibrium solid-phase diagram for the Mo–C–H system, illustrating the transformation of solid phases as a function of input CH_4_ content. The diagram highlights the sequential formation of different molybdenum-containing phases, demonstrating the stability regions of Mo, Mo_2_C, MoC, and C under varying CH_4_ content at a given temperature. As seen from the diagram, there are five phase fields in the Mo–C–H system at 1000 K: Mo + Mo_2_C, Mo_2_C, Mo_2_C + MoC, MoC, and MoC + C. As CH_4_ content is raised to about 0.5 mol, Mo_2_C phase is increasingly formed and a single Mo_2_C is obtained in the range of 0.5–0.57 mole of CH_4_. MoC phase appears at higher CH_4_ contents, free C forms along with MoC above 1.19 mol CH_4_. Figure 2b illustrates the variations in the gas content as a function of input CH_4_ amount at 1000 K. The graph provides insights into the equilibrium distribution of different gas-phase species in the system, highlighting the relative abundance of key components. Among these, H_2_ appears to be the major species and has the highest partial pressure close to 1 atm. The other species, CH_4_, CH_3_, H, C_2_H_4_, and C_2_H_6_ are predicted to have much lower partial pressures. As the amount of CH_4_ increases above 0.5 mol, the partial pressures of hydrocarbon species slightly rise. The increase in the partial pressures of carbonaceous species when Mo_2_C is present can be attributed to the continuous decomposition of methane and the limited capacity of Mo_2_C to incorporate additional carbon atoms. Once the Mo_2_C phase is fully formed, further dissociation of CH_4_ leads to the formation of excess carbon-containing gas-phase species such as CH_3_, C_2_H_4_, and C_2_H_6_, which accumulate in the gas phase as they are no longer consumed in carbide formation. This phenomenon has also been observed in studies investigating methane decomposition over molybdenum carbide catalysts, where the limited carbon uptake capacity of the carbide led to an increase in hydrocarbon gas species in the gas phase [20]. Moreover, in-situ studies have demonstrated that molybdenum carbides can form dynamically under reactive environments, influencing the interaction between CH_4_ and metal surfaces, further affecting gas-phase composition [21]. The phase transitions occur due to the progressive carburization of molybdenum by methane decomposition. The system is modeled under thermodynamic equilibrium conditions in the Mo-CH_4_ system at 1000 K, where CH_4_ is introduced as the carbon source, and its interaction with molybdenum leads to the formation of carbide structures. The primary reactions governing these transformations are given in Equations 5–6. These reactions result in the sequential formation of Mo_2_C, MoC, and free carbon as a function of increasing CH_4_ amount.


(5) Mo(s)+12CH4(g)→12Mo2C(s)+H2(g)

(6) $${\text{Mo}}_{2}\text{C}(\text{s})+3{\text{CH}}_{4}(\text{g})\to 2\text{MoC}(\text{s})+\text{C}(\text{s})+6{\text{H}}_{2}(\text{g})$$

The thermodynamic model assumes that CH_4_ undergoes dissociation, producing intermediate hydrocarbon species and hydrogen gas. These gaseous species play a crucial role in the overall carburization mechanism, influencing the formation of molybdenum carbide phases at different pressure regimes. Some decomposition reactions of methane into solid carbon and hydrogen are described by Equations 7–9.


(7) $${\text{CH}}_{4}\to \text{C}(\text{s})+2{\text{H}}_{2}$$

(8) $${\text{CH}}_{4}\to {\text{CH}}_{3}+\text{H}$$

(9) $${\text{CH}}_{3}\to \text{C}(\text{s})+{\text{H}}_{2}$$

Thermodynamic calculations suggest that the MoC+C phase can be obtained as an end product at methane gas pressures higher than 1.18 mole. Furthermore, this compound undergoes significant decomposition at 1100 K, generating the gaseous species H_2_ and C_x_H_y_ [22]. However, based on the X-ray diffraction (XRD) findings, the experimental results confirm that the Mo_2_C phase was formed at 1000 K in the methane atmosphere. Thermodynamic approximations contribute to the thermochemistry of the system by calculations in closed systems under constant pressure and provide predictions for experimental studies. However, it should be considered that under experimental conditions, some of the reactant gas reacts with the powder, and some flows over the powder bed and is discharged through the furnace outlet. Due to this situation, there may be a discrepancy between the thermodynamic approach and the experimental results.

### 3.2. DSC/TGA analysis of AMT

**Figure 3:**
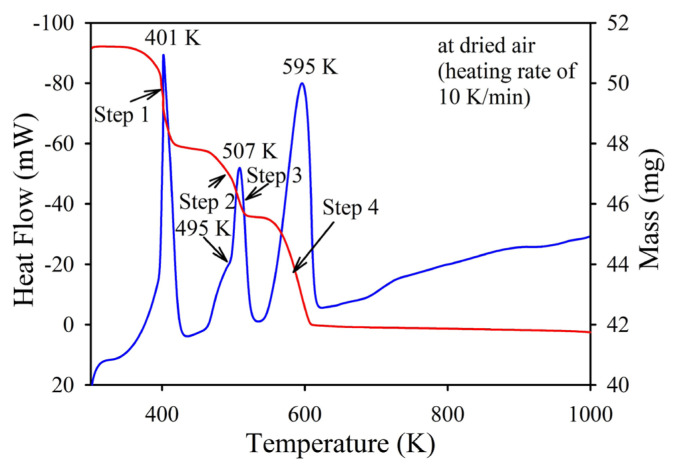
DSC/TGA curves of AMT powder in air.

Figure 3 shows the DSC/TGA curves of the AMT powder used. The TGA curve indicates that the mass of the sample powder decreases as the temperature is raised from 360 K to 610 K. The mass loss occurs in four steps, which are marked with arrows, in this temperature range. There are also four DSC peaks associated with each step. The first step with a mass loss of 3.1% and an endothermic peak at 401 K occurs in the temperature range 360–420 K. This step was attributed to removing some of the weakly bonded water molecules from AMT [23]. The second and third steps, with the corresponding endothermic peaks at 495 K and 507 K, were observed between 460 K and 520 K. It was reported that the endothermic peaks were ascribed to the evolution of intercalated crystalline water and ammonia [24]. Since there is a mass loss at this stage, as shown in the figure, it is plausible to suggest that some of the species formed are removed from the solid to the gas phase. The fourth step appears to be at temperatures between 540 K and 610 K, where an endothermic peak is observed at 595 K. The last peak is reported to be assigned to the loss of remaining crystalline water and ammonia [24]. Beyond 610 K, the mass remains almost constant at 81.79%, corresponding to a total mass loss of 18.21%. This mass loss is close to the theoretical value (18.45%) calculated in accordance with Equation 10, which expresses the decomposition of AMT into solid MoO_3_ and the gaseous reaction products NH_3_ and H_2_O. This suggests that NH_3_ and H_2_O species are removed from AMT during heating in air. The reaction expressed in Equation 10 is given.


(10) $${\left({\text{NH}}_{4}\right)}_{6}{\text{MO}}_{7}{\text{O}}_{24}.\;4{\text{H}}_{2}\text{O}\to 7{\text{MoO}}_{3}(\text{s})+6{\text{NH}}_{3}↑+7{\text{H}}_{2}\text{O}↑$$

### 3.3. Synthesis of Mo_2_C

The masses of AMT held in the tubular furnace in Ar at 900 and 1000 K for 2 h were measured to be 81.44% and 78.60%, which are below the theoretical mass (81.55%) of the conversion of AMT to MoO_3_. These results indicate that under the conditions studied, there is a slight Mo loss into the gas phase at 900 K and 1000 K. At 800 K for 4.5 h, it was 81.55%, which is the same as the theoretical value, suggesting no Mo loss to the gas phase. Hence, the AMT sample held at 800 K for 4.5 h was used for the synthesis of metallic Mo and Mo_2_C.

**Figure 4:**
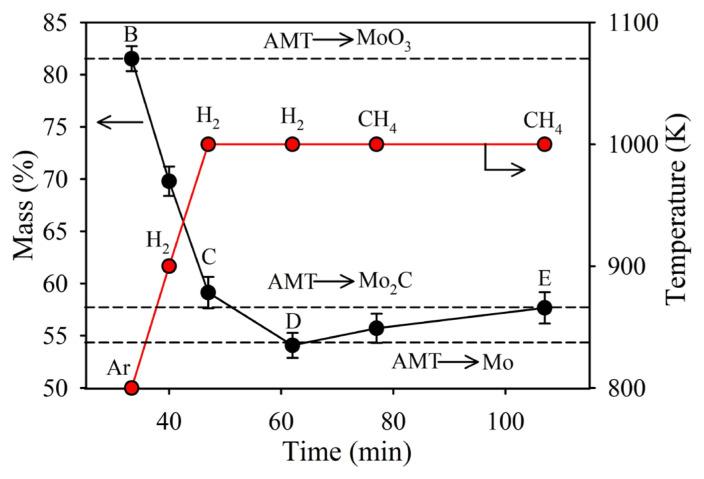
Variation in the AMT mass with temperature and time in Ar, H_2_ and CH_4_ atmospheres. The red line represents the temperature-time profile.

Figure 4 shows the variation in AMT mass with time under Ar, H_2_, and CH_4_ atmospheres represented by the black curve. The red curve illustrates the variation in temperature with time. The points on the curves represent the type of gaseous atmosphere. The AMT precursor (NH_4_)_6_Mo_7_O_24_.4H_2_O undergoes thermal decomposition under an Ar atmosphere as the temperature reaches 800 K. After heating in Ar to 800, the mass is measured to be 81.44% at Point B, which is close to the theoretical mass (81.55%) expected for the conversion of AMT to MoO_3_. This stage marks the formation of MoO_3_, a crucial intermediate phase before reduction. The decomposition process releases volatile ammonium species, leaving behind the molybdenum oxide as the solid phase. At Point C, when the system is under H_2_ flow, the solid curve in Figure 4 shows a gradual mass decrease as the sample is heated to 1000 K due to the reduction of MoO_3_ to MoO_2_ and Mo. After 15 min of H_2_ flow at 1000 K, the mass is stabilized at 54.08%, which closely matches the theoretical value for complete AMT to Mo conversion (54.36%), as indicated by the horizontal dashed line. The H_2_-reduction of MoO_3_ can be expressed by Eq. 12. The sample is held at 1000 K in H_2_ for 15 min at Point D, leading to the complete reduction of Mo oxides to metallic Mo. Hence, the mass loss is attributed to the removal of oxygen from MoO_3_. This means that Mo is available in its pure form before carburization in the next stage. The mass increases to 57.67% at Point E within 45 min of CH_4_ flow at 1000 K, which is close to the theoretical value for AMT to Mo_2_C conversion mass (57.74%). This result indicates that Mo gained mass by C uptake. This final transformation corresponds to the carburization of Mo to form Mo_2_C, expressed by Eq. 14. This step marks the completion of the reaction sequence, where Mo reacts with CH_4_ to form molybdenum carbide (Mo_2_C) as the dominant phase. The successful conversion of Mo to Mo_2_C, marking the completion of the reaction sequence, was confirmed by XRD data. The reaction equations mentioned are given in the following section after the discussion of the XRD results.

The reaction kinetics and phase transition dynamics of Mo_2_C formation involve sequential reduction and carburization steps, which are strongly dependent on temperature, gas composition, and precursor reactivity. The reduction of MoO_3_ to Mo occurs through an intermediate MoO_2_ phase, as confirmed in previous studies on the thermochemical transformation of molybdenum oxides [25,26]. The complete reduction to metallic Mo is a critical step before carburization, where CH_4_ decomposition facilitates carbon incorporation into the Mo lattice, leading to Mo_2_C formation. Thermodynamic calculations predict that the reaction follows a stepwise reduction pathway, in agreement with previous reports detailing Mo_2_C synthesis from molybdenum oxides [27].

**Figure 5:**
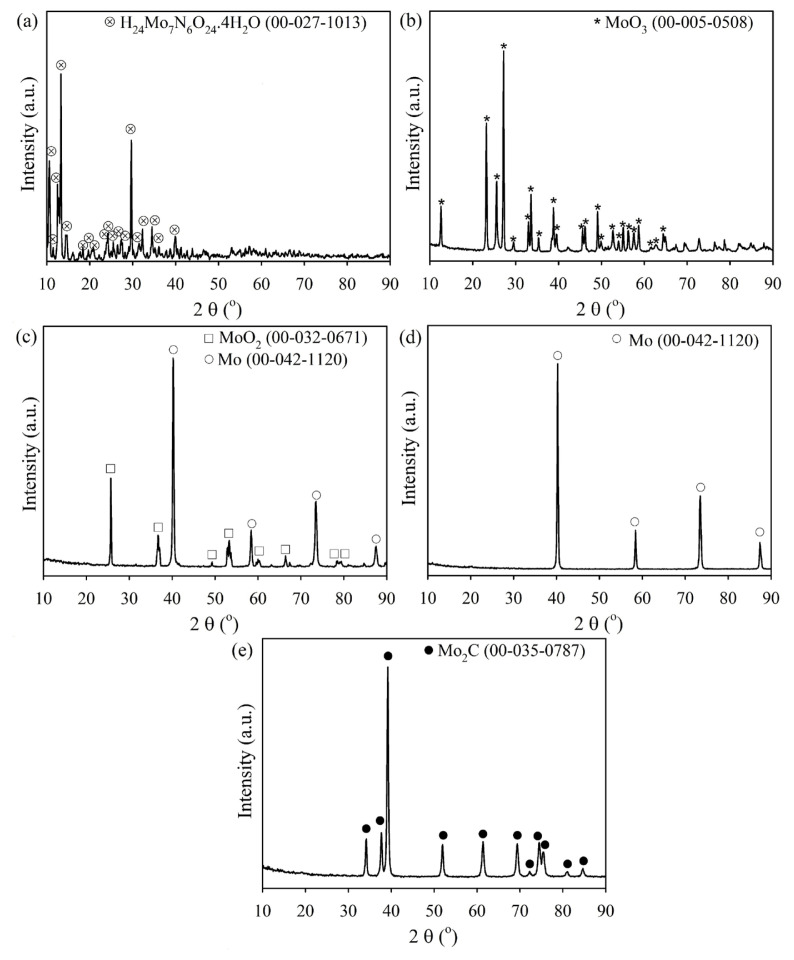
XRD patterns of AMT (a) and the products obtained at the conditions marked (b) Heating in Ar to 800 K – AMT to MoO_3_ conversion (point B), c) Heating in H_2_ from 800 K to 1000 K – Formation of MoO_2_ and Mo (point C) d) Isothermal holding in H_2_ at 1000 K – Complete reduction to metallic Mo (point D), e) Isothermal holding in CH_4_ at 1000 K to complete carburization to Mo_2_C (point E)

Figure 5 shows the XRD patterns of the AMT powder and the products derived from it. Pattern a presents the characteristic peaks of AMT with a monoclinic crystal structure reported in powder diffraction files (PDFs) no. 00-027-1013 [28]. Pattern b belongs to the product obtained when the AMT was heated to 800 K in Ar. All the diffraction peaks correspond to MoO_3_ with an orthorhombic crystal structure (PDF 00-05-0508). Pattern c reveals that MoO_3_ is reduced to a mixture of MoO_2_ and Mo phases during heating to 1000 K. After 15 min of holding at 1000 K in H_2_, the product exhibits only Mo peaks (pattern d). Subsequent holding at 1000 K for 45 min under a CH_4_ atmosphere yields pure-phase Mo_2_C, as revealed by pattern e. The diffraction peaks in this pattern are assigned to closed-packed hexagonal Mo_2_C as the interplanar spacing, and the intensities of the peaks agree with those listed in PDFs 035-0787. For example, the first three peaks at 2θ = 34.176°, 2θ = 37.712°, and 2θ = 39.197° are assigned to the (100), (002), and (101) crystal planes, respectively. The calculated interplanar spacings (0.2621 nm, 0.2383 nm, and 0.2296 nm) and their relative intensities (18, 23, 100) match well with the published standard values (0.2608 nm, 0.2367 nm, 0.2285 nm, 20, 25, 100). The XRD patterns obtained in this study confirm the sequential phase transformation from AMT to Mo_2_C. The diffraction peaks observed for Mo_2_C closely match those reported in previous studies, for example, the peaks at 2θ = 34.3°, 37.9°, and 39.5°, which correspond to the (100), (002), and (101) planes of Mo_2_C [29]. These findings align with studies reporting Mo_2_C synthesis using CH_4_/H_2_ gas mixtures, where similar peak positions and relative intensities were observed [25]. Furthermore, phase purity is a crucial aspect of Mo_2_C synthesis. Some studies have reported the formation of secondary phases such as MoO_2_ and MoC when reaction conditions deviate from thermodynamically favorable regions [30]. However, the present study confirms that the optimized reaction conditions successfully yield phase-pure Mo_2_C, as indicated by the absence of MoO_2_ or MoC peaks in the XRD patterns. These results demonstrate that the proposed synthesis method effectively achieves high purity comparable to established Mo_2_C synthesis techniques.

Based on the XRD patterns, it is plausible to suggest the primary reaction steps as follows: ${\left({\text{NH}}_{4}\right)}_{6}{\text{Mo}}_{7}{\text{O}}_{2}\overset{Ar}{⃗}{\text{MoO}}_{3}\overset{H_{2}}{⃗}>{\text{MoO}}_{2}\overset{H_{2}}{⃗}\text{Mo}\overset{CH_{4}}{⃗}{\text{Mo}}_{2}\text{C}$. The overall reactions leading to the MoO_3_, MoO_2_, Mo, and Mo_2_C phases are expressed by Eqs.11–14.


(11) $${\left({\text{NH}}_{4}\right)}_{6}{\text{Mo}}_{7}{\text{O}}_{24}.\;4{\text{H}}_{2}\text{O}\to 7{\text{MoO}}_{3}+6{\text{NH}}_{3}+7{\text{H}}_{2}\text{O}$$

(12) $${\text{MoO}}_{3}+{\text{H}}_{2}\to {\text{MoO}}_{2}+{\text{H}}_{2}\text{O }\;\;\Delta {{\text{G}}^{\text{o}}}_{\text{r}}=-104140\;\text{J}/\text{mol at }1000\;\text{K}$$

(13) $${\text{MoO}}_{2}+2{\text{H}}_{2}\to \text{Mo}+2{\text{H}}_{2}\text{O }\;\;\Delta {{\text{G}}^{\text{o}}}_{\text{r}}=20217\;\text{J}/\text{mol at }1000\;\text{K}$$

(14) Mo+12CH4→12Mo2C+H2         ΔGor=-38885 J/mol at 1000 K

where ΔG^o^_r_ is the Gibbs’ free energy change of the reaction. Reactions expressed by Equations (12) and (14) are thermodynamically favorable owing to the negative ΔG^o^_r_ values. ΔG°_r_ value for Equation 13 is positive at 1000 K, suggesting that the reaction is not favorable. However, the reduction of MoO_2_ by H_2_ is possible when the partial pressure of water is relatively low [31].

A complex series of reactions during CH_4_ decomposition leads to the formation of intermediate hydrocarbons (e.g., CH_3_, CH_2_, CH, and C) that gradually become hydrogen-poor [32]. The reaction between C adsorbed on the particle surface and the Mo particle may yield Mo_2_C. Rather than producing CO_2_, reactions expressed by equations (11–14) and thermodynamic calculations do not yield CO_2_ as a product, but gaseous products such as NH_3_, H_2_O, and H_2_. Furthermore, CH_4_ was introduced to the furnace after single-phase elemental Mo was used to avoid CO_2_ formation during the carburization process.

**Figure 6:**
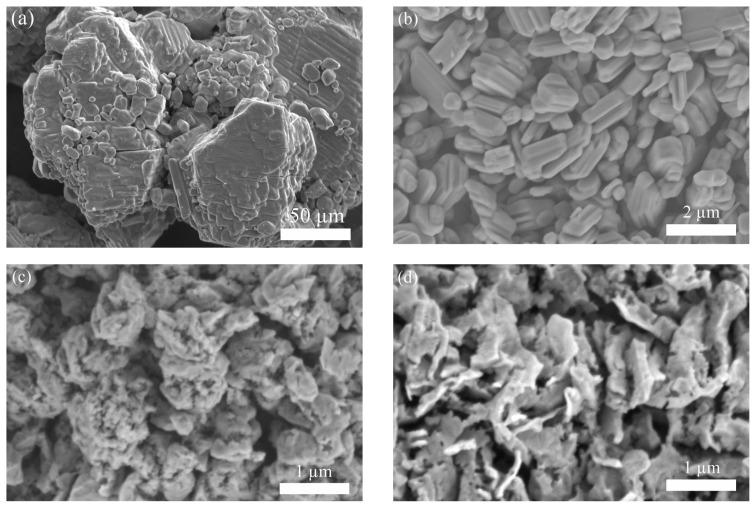
SEM images of (a) the AMT and (b) MoO_3_, (c) Mo plus MoO_2_, (d) Mo and (e) Mo_2_C powders.

Figure 6a-e shows SEM images of the AMT, MoO_3_, Mo+MoO_2_, Mo, and Mo_2_C powders, respectively. The AMT powder mostly consists of large particles (>50 μm) with highly stacked features (image a). When AMT is converted to MoO_3_, finer particles appear, as seen in image b. The morphology is disturbed, as revealed by the rough surfaces when MoO_3_ is partially reduced to Mo (image c). The Mo powder typically consists of platelets with thicknesses ranging from approximately 160 to 600 nm (image d). After the carburization process, the Mo_2_C product has a morphology substantially similar to that of Mo, as revealed by image e. A finer morphology (thickness ≈100 nm) is observed as the transformation takes place in the path of AMT (monoclinic) > MoO_3_ (orthorhombic), MoO_2_ (monoclinic) >Mo (b.c.c.), and Mo_2_C (hexagonal). The changes in morphology and size can be attributed to cracks, pores, fissures, and delamination due to vacancy coalescence and stresses formed during the volumetric changes induced by the structural transformations. The densities are 2.50, 4.69, 6.47, 10.28, and 8.9 g/cm^3^ for AMT, MoO_3_, MoO_2_, Mo, and Mo_2_C, respectively. The AMT to MoO_3_, MoO_3_ to MoO_2_, and MoO_2_ to Mo transformations all create tensile stresses in the transformed layers when the ratio of the volume of the product to that of the parent phase is less than unity. Tensile stresses can fragment the particles into smaller ones. The conversion of Mo to Mo_2_C creates compressive stress in Mo_2_C (volumetric ratio > 1), which may lead to delamination of the platelets. Some defects in the Mo_2_C particles are possibly inherited from the thermal decomposition of AMT and oxide reduction processes. Rapid reduction/carburization occurs due to direct contact of the reactive gases (H_2_ and CH_4_) with fresh parent surfaces through surface cracks and through highly porous, cracked layers of Mo and Mo_2_C. The SEM images presented in this study illustrate the morphological transformations occurring throughout the synthesis, from AMT to Mo_2_C. The formation of intermediate phases, such as MoO_3_ and MoO_2_, results in notable changes in particle morphology, as observed in previous studies on Mo_2_C synthesis [7]. The transition from MoO_3_ to Mo results in the formation of platelet-like structures, which are retained upon carburization to Mo_2_C. This is consistent with previous findings, where Mo_2_C synthesized via C_3_H_8_ carburization exhibited similar morphologies [33]. Additionally, carburization conditions significantly impact the particle size and surface roughness of Mo_2_C. Studies indicate that an increase in CH_4_ concentration leads to the development of finer Mo_2_C grains, whereas lower CH_4_/H_2_ ratios promote the formation of larger, more faceted particles [27]. The SEM observations in the present study suggest that the selected synthesis conditions lead to a morphology that aligns well with those reported in previous literature.

Previous studies have explored various approaches to Mo_2_C synthesis, including carburization using different carbon precursors, thermochemical reduction, and vapor-phase synthesis. While traditional methods such as solid-state carburization require high temperatures (~1273 K) and long reaction times (several hours), alternative techniques, including gas-phase carburization using CH_4_/H_2_ mixtures, have demonstrated reductions in temperature and reaction duration [34] However, many of these approaches still result in the formation of CO_2_ as a byproduct, contributing to environmental concerns [27]. In contrast, the present study introduces a single batch gas-phase synthesis approach using AMT as the precursor, which enables Mo_2_C formation at 1000 K in a significantly shorter reaction time (~1 hour). Compared to other methods, this approach eliminates CO_2_ emissions by leveraging CH_4_ decomposition into hydrocarbon species instead of CO/CO_2_-producing pathways. Additionally, thermodynamic calculations confirm that the reaction pathway follows a stepwise reduction and carburization process, aligning with previous theoretical studies [35] This approach also ensures a highly controlled synthesis environment, yielding uniform and high-purity Mo_2_C platelets. Regarding energy efficiency, the ability to synthesize Mo_2_C at a lower temperature and within a single batch minimizes energy consumption, making the process more sustainable than conventional high-temperature carburization techniques [34]. Furthermore, in comparison to solution-phase synthesis of Mo_2_C, which involves complex reaction media and additional post-processing steps [29]. The current method offers a direct and scalable approach to Mo_2_C production, making it suitable for industrial applications.

## 4. Conclusions

Mo_2_C was synthesized in a single batch under the guidance of thermodynamic predictions by strategically switching the gas atmosphere. Thermodynamic calculations carried out in Mo-O-H and Mo-C-H systems predict that Mo and Mo_2_C syntheses are feasible at 1 atm and 800–1000 K and 1000 K, respectively. The initial stage of the reaction was conducted under an argon environment, which was then switched to a hydrogen (H_2_) atmosphere at 800 K to obtain Mo. Subsequently, the gas flow was switched from H_2_ to methane at 1000 K for Mo_2_C synthesis. The changes in crystal structures during the transformation follow the path of AMT (monoclinic) > MoO_3_ (orthorhombic) > MoO_2_ (monoclinic) > Mo (b.c.c.), and Mo_2_C (hexagonal). Fine Mo_2_C particles with a thickness of 0.1 μm were obtained from very coarse AMT particles (>50 μm). The study demonstrated the feasibility of this straightforward single batch synthesis method for obtaining pure-phase Mo_2_C powder avoiding carbon dioxide formation, which is an important consideration for the development of eco-friendly and energy-efficient material processing techniques in terms of synthesis reaction time.
